# Quality Evaluation of Entrepreneurship Education in Chinese Medical Colleges–From the Perspective of Student Cognition

**DOI:** 10.3389/fpsyg.2020.01093

**Published:** 2020-06-17

**Authors:** Yuhui Li, Wei Shen, Yijun Lv

**Affiliations:** ^1^School of Innovation and Entrepreneurship, Wenzhou Medical University, Wenzhou, China; ^2^School of Innovation and Entrepreneurship, Zhejiang Wanli University, Ningbo, China

**Keywords:** medical, quality, evaluation, entrepreneurship education, student

## Abstract

The evaluation of the effectiveness of entrepreneurship education has become a key issue in improving the quality of entrepreneurship education. The quality of entrepreneurship education was empirically analyzed through a questionnaire survey conducted within 70 medical colleges and universities in China and 16,660 valid questionnaires were obtained. The datasets were processed with a classic analysis tool, SPSS. Several findings were revealed by the research. The popularity of entrepreneurship courses in China’s medical schools was low, due to reasons such as: the obvious characteristics of fragmentation in curriculum design the entrepreneurship practice for medical students being far from open and not effectively integrated with the market trend; the current policies in China not providing additional support for medical entrepreneurship and the lack of funds, which is the main obstacle for medical students who owned start-ups; and the teacher-student collaboration not being an important enough vessel to improve the quality of entrepreneurship education. It is recognized in this paper that, in the future, medical schools in China should build an individualized and diversified medical entrepreneurship education curriculum system, strengthen the openness of medical students’ entrepreneurship practice, build a multi-channel financial support platform, and create a major-innovation integration mechanism.

## Introduction

From the perspective of epistemology, education evaluation is used to generate factual and objective judgments on education. Whether a judgment is deemed correct is verified by practice, based on whether subjective cognition conforms to objective criteria. Education, as a social phenomenon, not only needs the factual judgment but also needs to be judged on its value. The criterion for the value judgment lies in whether it satisfies the needs of human development and social development.

The concept of evaluation was first proposed by American scholar Ralph Tyler, who was honored as the father of educational evaluation. According to Tyler (2014), the evaluation process is essentially a process of determining the extent to which the curriculum and syllabus actually achieve educational goals. However, educational goals essentially refer to the changes that occur, that is, the goal to be achieved is some kind of desired change expected to be produced in the student behavior pattern. Therefore, evaluation is a process of determining the degree of actual change in behavior. Scholars with different perspectives are bound to have different reflections regarding the concepts of educational evaluation. Representatives such as Cronbach (1983) believed that educational evaluation is necessary to make decisions about educational programs, collecting and using information. Stenhouse (1975) defined evaluation as an evaluation of advantages/shortcomings or value, or an activity that has both a description and a judgment. It focused on judging the benefits of educational activities, educational processes, and educational outcomes, to examine if they have value. The Joint Committee on Standards for Educational Evaluation (JCSEE) established a comprehensive definition of educational evaluation in 1981, followed by the most recent revision in 2011, arguing that educational evaluation is a process providing a basis for educational decision-making after investigation of educational goals and its advantages, disadvantages, and value judgments (Joint Committee on Standards for Educational Evaluation [JCSEE], 2011). In a word, it states that education evaluation is used to continuously improve educational decision-making and meet the needs for human development and social development using a series of scientific means to judge the value of educational practice, educational processes, and educational results based on a certain educational value judgment standard and goal. The education evaluation mainly includes process evaluation and impact evaluation.

As a sub-study field of educational evaluation, evaluation of entrepreneurship education has been explored from different angles. For instance, Lundström and Stevenson (2005) evaluated entrepreneurship education in colleges and universities via strategic planning. Based on educational practice, Vesper and Gartner (1997) proposed a Seven-Factor Evaluation Method covering entrepreneurship courses, textbooks, school influence, enrollment rate of entrepreneurship education, graduate self-employment rate, survival rate of graduate entrepreneurial projects, and related activities of college teachers’ entrepreneurial activities. Researchers have a long history concentrating on how entrepreneurship education impacts on students. Wei X. et al. (2019) tested it on Multiple Mediation Model, Wei J. et al. (2019) constructed an interpretive structure model, and Wang et al. (2019) examined entrepreneurship education interaction with other domains. Since entrepreneurship education is a dynamic education system where theoretical construction and practical operation go in tandem, the evaluation of entrepreneurship education should follow the model of summative evaluation and formative evaluation. Based on such a theoretical perspective, Fayolle et al. (2006) proposed that the evaluation of entrepreneurship education mainly includes the paradigm of process factor evaluation and impact evaluation. Process factor evaluation is the evaluation for educators of the status of each element that constitutes entrepreneurship education (Henry et al., 2004). The process elements of entrepreneurship education include entrepreneurship course, entrepreneurship practice, entrepreneurship activities (entrepreneurship contest), investment in entrepreneurship education (policy support), and collaboration between teachers and students (entrepreneurship activities or entrepreneurship projects carried out by teachers and students together), which have a positive impact on entrepreneurship education (Henry et al., 2004). Impact evaluation is an assessment of the effect of self-changes after receiving the entrepreneurial education. It is tested by the changes it brings to the education receivers, including cognitive, emotional, and behavioral changes (Pedrini et al., 2017). In terms of the current research situation in China, scholars mainly follow the paradigm of process evaluation and impact evaluation in research on quality evaluation of entrepreneurship education (Xu et al., 2018). Some scholars (Ge and Liu, 2014) introduced the Context-Input-Process-Product (CIPP) education evaluation model into capability evaluations of entrepreneurship education, constructing the quality evaluation system from four corresponding aspects: context evaluation, input evaluation, process evaluation, and product evaluation.

Through literature review, it can be found that at the current stage, domestic and foreign scholars have focused on the quality evaluation of entrepreneurship education (EE) within comprehensive universities. Nevertheless, such research concentrating on medical colleges is still inadequate, and is where the interest of this paper was sourced. What is the quality of EE among medical schools in China? What is the effect of EE elements in the process of education practice? How could the quality of EE in China be improved? These problems are what this study tried to answer. The study established hypotheses to be tested based on the theoretical background of EE process elements. Multiple linear regression analysis and questionnaires delivered to students in 76 domestic medical schools were subsequently combined to investigate the proposed hypotheses. Statistical results were analyzed and discussed with the comparison to previous studies and implications for future research, to reflect the current status and lack of EE in domestic medical schools. Eventually, standing on the findings of this paper, implications and suggestions for practitioners and researchers were provided.

The contribution of this study was mainly manifested in three aspects. First, it filled the missing academic part through an empirical study focused specifically on medical schools in China. The result of research into entrepreneurship education has had beneficial effects within Chinese academia, but few scholars have paid attention to such research in medical schools. Second, based on a large amount of sampled data, this paper used a variety of analysis methods to make the conclusion more convincing. It aimed to overcome common problems that have occurred in the evaluation research of entrepreneurship education in China, such as inadequate sample sizes and lack of persuasion. Third, this paper proposed some targeted implications and suggestions, which could help medical schools to improve the quality of entrepreneurship education.

## Theoretical Background and Hypotheses Establishment

### Entrepreneurship Course

A curriculum is the core element of school education. It is the main carrier of school education objectives, education value embodiment, and syllabus implementation. As an important vessel of entrepreneurship talents training, entrepreneurship courses play an irreplaceable role in entrepreneurship knowledge impartment and entrepreneurship skills cultivation. Esmi et al. (2015) designed a set of entrepreneurship courses covering various needs of all parties. Through the effective integration and connection of direct teaching, teacher-student interaction, and practical operation, the teaching model of entrepreneurship courses was constructed to cultivate students’ entrepreneurial skills. Through empirical research, Azila-Gbettor and Harrison (2013) found that the entrepreneurship course in polytechnic universities suffered from a lack of applicability, which meant the impartment of entrepreneurship knowledge was unable to meet the needs of students. Thus, the graduates were unable to utilize the knowledge acquired when they obtained the off-campus entrepreneurship resources, and that is why low satisfaction rates regarding entrepreneurship education was expected in the polytechnic universities. Heinrichs (2016) designed the curriculum content with entrepreneurship cases, making up for the traditional entrepreneurship course which only concentrated on the accumulation of theoretical knowledge with ignorance in the practical experience. Through the sharing of entrepreneurship cases such as successful start-ups, the satisfaction of entrepreneurship education was effectively improved. Thus, the first hypothesis can be set as:

*H1:* Entrepreneurship Courses provide a positive influence on entrepreneurship education evaluation.

### Entrepreneurship Contest

An entrepreneurship contest is also referred to as a subject contest. Hu et al. (2018) claimed that subject contest has become a crucial platform to generate all-round development for the students, a significant carrier for entrepreneurship education reform in China, and a key link to promote the combination of Industry-University-Research. Jin et al. (2017) believed that the entrepreneurship contest in Chinese style was an all-round exercise aiming to cultivate innovative spirit and the formation of practical ability through literature review, independent topic selection, innovative research, and team cooperation. To overcome various difficulties in research, certain guidance was offered by teachers throughout the contest. Fan et al. (2016) reported that China’s entrepreneurship contest relied on different platforms at university, provincial, and national levels, which constructs a three-dimensional subject contest system for different grades such as basic level, intermediate level, and innovation level. Song and Yang (2016) introduced practice results which have demonstrated that an education model with subject contest at its core has a significant impact on innovation and entrepreneurship education. It is reasonable to establish the second hypothesis.

*H2:* Entrepreneurship Contest provides a positive influence on entrepreneurship education evaluation.

### Entrepreneurship Practice

Entrepreneurship practice is a vital factor affecting entrepreneurship willingness and skills. It is a boost to the promotion of entrepreneurship ability. Jena (2020) investigated the cognition, emotion, and behavior of Indian college students on entrepreneurship education by random sampling and discovered that entrepreneurship practice had a significant positive effect on entrepreneurship intention. Dodescu et al. (2014) took entrepreneurship projects as the starting point to build a partnership of mutual support between entrepreneurship knowledge and practice by combining qualitative research and quantitative research. Entrepreneurship practice not only strengthened entrepreneurship skills, but also cultivated entrepreneurship spirit. In addition, it also increased the entrepreneurship willingness of potential entrepreneurial students. Zhao et al. (2018), based on the practical experience of entrepreneurship education in China, believed that entrepreneurship education helped improve student’s innovation and entrepreneurship ability, and that this opportunity should be taken to promote the development of China’s education industry. The third hypothesis can be described as:

*H3:* Entrepreneurship Practice provides a positive influence on entrepreneurship education evaluation.

### Policy Support

Policy support plays an important role in enhancing the willingness of entrepreneurship in the actual process of entrepreneurship. The greater the policy support is, the stronger entrepreneurship intention students can have. Hoppe (2016) found that the Swedish government’s preferential policies for entrepreneurship had significantly improved the business environment in Sweden, which, to a certain extent, encouraged people’s intentions to start innovative businesses. Zhao and Ziedonis (2012) carried out an investigation, showing that the United States government offered certain financial supports, such as allowances and/or subsidies for technology-oriented start-ups. Through the follow-up survey of 241 technology-oriented start-ups in the United States, it transpired that start-ups had been frequently funded by the federal or state governments and those that had been supported by tax refund policies exhibited a relatively high survival rate. Subsequently, the high survival rate of these start-ups helped to increase market confidence, not to mention the entrepreneurship intention. This results in our fourth hypothesis.

*H4:* Policy Support has an advantageous impact on entrepreneurship education evaluation.

### Co-entrepreneurship of Teachers and Students

Co-entrepreneurship describes the collaboration of teachers and students. It is conducive to the commercialization of innovative, scientific, and technological achievements. Kliewe et al. (2016) discovered that University-Enterprise Cooperation (UBC), as a basic form of cooperation between teachers and students, was supported and guided by teachers and enterprises in practice to effectively help the commercialization of scientific and technological achievements. It was easier to improve the effect of entrepreneurship education through such a formality. Through empirical comparison, San-Martín et al. (2019) found that teachers, as entrepreneurship role models, significantly increased student entrepreneurship willingness, cognition, and ability. Moreover, the difficulty of entrepreneurship was reduced and the success rate was improved. Out of these theoretical analyses, it is reasonable to hypothesize that:

*H5:* Collaboration between Teachers and Students has a positive influence on entrepreneurship education evaluation.

## Materials and Methods

### Sample and Measurement

A questionnaire survey was used to evaluate the quality of medical college entrepreneurship education. An online invitation with an exclusive QR code was distributed to students within 70 medical specialized universities across China (Hong Kong SAR, Macau SAR, and Taiwan were excluded), outlining the brief purpose of the study and an incentive of CNY5 which could be earned once they extract the QR code to enter the survey, verify the student identity, then validly complete the task. The survey was operated in the form of an online questionnaire where the data could be accumulated and statistically calculated directly. The questionnaire contained 37 items; each item was measured by a 5-point Likert scale. Summarized from a statistic report, 90.24% of collected questionnaires were determined to be valid because certain preset discard rules were triggered through means such as studentship verification failure, being fraudulent with the incentive, important information being missing, etc.

The sample contained five “double first-class” universities (top class universities) with 465 students, fifty-five ordinary universities and colleges with 15170 students, nine independent colleges and universities with 1007 students, and one private college with 18 students, accounting for 2.79, 91.06, 6.04, and 0.01%, respectively, in sample quantity. No higher vocational colleges were involved. The distribution met the trend of statistics from the Ministry of Education of PRC, demonstrating that the survey was properly spread.

The geographical distribution of sampled students was shown in Table 1. These students were randomly invited to avoid any unscientific factors that could result from manual division intervened sampling strategies, such as stratification of clustering, which could introduce biased results, especially considering the enormous and complex differences of the culturally-, geographically-, and socio-environmentally influenced habitants of China.

**TABLE 1:** Geographical distribution of sampled students.

**Eastern area**	**Number**	**Central area**	**Number**	**Western area**	**Number**
Beijing	0	Shanxi	2727(16.37%)	Szechuan	403(2.42%)
Tianjin	287(1.72%)	Inner Mongolia	99(0.59%)	Guizhou	2(0.01%)
Hebei	741(4.45%)	Jilin	3(0.02%)	Yunnan	4(0.02%)
Liaoning	677(4.06%)	Heilongjiang	3744(22.47%)	Tibet	0
Shanghai	0	Anhui	456(2.73%)	Shaanxi	100(0.60%)
Jiangsu	644(3.87%)	Jiangxi	379(2.28%)	Gansu	1(0.01%)
Zhejiang	955(5.73%)	Henan	507(3.04%)	Qinghai	0
Fujian	2986(17.92%)	Hubei	28(0.17%)	Ningxia	1(0.01%)
Shandong	1222(7.35%)	Hunan	0	Xinjiang	0
Guangdong	392(2.35%)	Guangxi	0		
Hainan	299(1.79%)	Chongqing	3(0.02%)		
Eastern total	8203(49.24%)	Central total	7946(47.70%)	Western total	511(3.07%)

It is recognized that forensics, science, and engineering are the majors directly related to medical education. It was found in the survey that the students’ majors were medicine (78.10%), science (9.80%), engineering (3.50%), forensics (0.43%), philosophy (0.13%), economics (0.53%), education (0.46%), literature (0.51%), history (0.01%), agriculture (0.40%), military (0.01%), management (6.50%), and art (0.12%).

### Validity Analysis

Under the preliminary calculation of Exploratory Factor Analysis for the original questionnaire, the Kaiser-Meyer-Olkin (KMO) measure was 0.982, which was greater than 0.8, indicating a plausibility of factor analysis. The χ2 of Bartlett’s Test of Sphericity (Tobias and Carlson, 1969) was 836195.261, the degree of freedom was 666, and the *P*-value was less than 0.001. The significance standard was reached, the independence hypothesis of each variable was not established, and common factors among related matrices of the parent group existed, which were suitable for factor analysis. Principal Component Analysis and Maximum Variance Rotation Method were used to extract factors with an eigenvalue greater than 1.4 and factor load greater than 0.5. The common values of all items in the questionnaire were located at the interval from 0.722 to 0.949 after extraction, indicating a good commonality. Three factors could be extracted, and the cumulative variance contribution rate reached 73.16%, significantly higher than 60%. It could be considered that the variance contribution rate was high and the questionnaire structure validity was satisfying.

### Reliability Analysis

The reliability α coefficient of 37 items was 0.981, which indicated that the homogeneity reliability was highly ideal. The reliability value of each factor was greater than 0.7, indicating that the questionnaire has a high reliability. The reliability of each factor and total reliability were presented in Table 2.

**TABLE 2:** Cronbach’s α of factors.

**Factor**	**Cronbach’s α**	**Items**
1	0.726	3
2	0.984	30
3	0.977	4
Total	0.981	37

According to the Exploratory Factor Analysis, three factors were extracted: “individual condition scale,” “entrepreneurship education quality evaluation scale,” and “teacher-student collaboration scale”. The individual condition scale included four original items in the questionnaire: the family’s social resources, entrepreneurship situation in the surroundings, social environment for entrepreneurship, and personal ability. Since the standard deviation of a question involved was close to 0, which meant that the answers were highly consistent, the remaining three were retained. The higher score was offered, the better the individual situation could be. In this study, the internal consistency coefficient of individual situation was 0.726. Entrepreneurship education evaluation included 30 test questions, then four sub-factors were further extracted after a second exploratory factor analysis, including entrepreneurship course, contest projects, entrepreneurship practice, and policy support. After testing, the scale structure was proved to be clear. The cumulative variance interpretation rate was 84.90% and each item was loaded on the corresponding factor. In this study, the consistency coefficients of all dimensions in the scale were 0.934, 0.960, 0.957, and 0.965, respectively. The teacher-student collaborating create scale included 5 test questions. Similar to the situation of the individual condition scale, one question was deleted as the standard deviation of which was close to 0. In this study, the internal consistency coefficient was 0.977.

## Results

### Individual Condition Scale Output

The descriptive statistical evaluation of entrepreneurship education under individual conditions were shown in Tables 3, 4.

**TABLE 3:** Frequency analysis of individual conditions of sampled students.

**Primary indicator**	**Secondary indicator**	**Likert point**					**Mean**
		**5**	**4**	**3**	**2**	**1**	
Individual conditions	D1	973 (5.8%)	1879 (11.3%)	5409 (32.4%)	4456 (26.7%)	3499 (23.7%)	2.49
	D2	1076 (6.5%)	2942 (17.6%)	8212 (49.3%)	2767 (16.6%)	1663 (10.0%)	2.94
	D3	713 (4.3%)	1469 (8.8%)	7097 (42.6%)	4803 (28.8%)	2578 (15.5%)	2.58

*D1: Colleagues or friends have started their own businesses within the past year. D2: Business policies and the environment in your province are favorable. D3: You believe that you have enough knowledge, skills, and experience to start a business.*

**TABLE 4:** Score of entrepreneurship education under different individual conditions.

**Factors**	**Groups**	**Quality evaluation**	
		**Mean**	**Standard deviation**
Gender	Male	3.67	0.922
	Female	3.75	0.841
Ethnics	Han	3.73	0.866
	Others	3.65	0.906
Only child	Positive	3.72	0.892
	Negative	3.73	0.854
Practice in school	Positive	3.87	0.837
	Negative	3.68	0.873
Post-graduation	Employment	3.71	0.857
	Further education	3.72	0.870
	Entrepreneurship	3.82	0.935
	Others	3.69	0.846
Entrepreneurial experience of immediate family members	Positive	3.83	0.850
	Negative	3.70	0.871
Grades	Top 25%	3.79	0.854
	Upper 25%	3.75	0.839
	Lower 25%	3.64	0.874
	Bottom 25%	3.55	0.997

The score of individual condition was generally low with an overall score of 2.67. Among them, the score of “colleagues and friends who have started a business within the past year” was the lowest. Spearman Correlation Analysis (Fieller and Pearson, 1961) was used to figure out that the quality evaluation of entrepreneurship education was significantly positively correlated with the entrepreneurship of individuals (*r* = 0.261, *P* < 0.001).

One-way analysis of variance was used to test whether factors like gender, ethnic group, being an only child, practice in school, destination after graduation, immediate family members’ entrepreneurial experience, and grades in college had any impact on the quality of entrepreneurship education. As what could be seen in Table 5, women gave a better quality evaluation of entrepreneurship education than men (*F* = 31.46, *P* < 0.001). The evaluation from the Han ethnic group was higher than the others (*F* = 8.21, *P* = 0.004). Being an only child had no significant impact on the evaluation (*F* = 0.35, *P* = 0.555). The participants of entrepreneurial innovation offered a higher quality than non-participants (*F* = 7.43, *P* = 0.006). Students who planned to start their own business after graduation evaluated the quality of entrepreneurship education more highly than those who did not plan to be involved in business (*F* = 3.63, *P* = 0.012). Students whose immediate family members had entrepreneurial experience rated a higher quality than those who had not (*F* = 59.99, *P* < 0.001). The higher the score, the higher the quality of entrepreneurship education (*F* = 41.75, *P* < 0.001).

**TABLE 5:** Evaluation of each element in entrepreneurship education.

**Primary indicator**	**Secondary indicator**	**Likert point**					**Mean**
		**5**	**4**	**3**	**2**	**1**	
Contest projects	Diversity	2467(14.8%)	4736(28.4%)	7107(42.7%)	1587(9.5%)	763(4.6%)	3.39
	Easy implementation	1999(12.0%)	3883(23.3%)	7899(47.4%)	1986(11.9%)	893(5.4%)	3.25
	Compatibility	2249(13.5%)	4214(25.3%)	7482(44.9%)	1863(11.2%)	852(5.1%)	3.31
	Improved ability	2681(16.1%)	5572(33.4%)	6670(40.0%)	1150(6.9%)	587(3.5%)	3.52
	Improved confidence	2765(16.6%)	5718(34.3%)	6549(39.3%)	1069(6.4%)	569(3.4%)	3.54
	Expended social network	3076(18.5%)	5976(35.9%)	6208(37.3%)	894(5.4%)	506(3.0%)	3.61
	Improved teamwork	3499(21.0%)	6443(38.7%)	5634(33.8%)	672(4.0%)	412(2.5%)	3.72
	Enhanced real entrepreneurship	3052(18.3%)	5811(34.9%)	6389(38.3%)	923(5.5%)	486(2.9%)	3.6
Course	Versatile teaching approaches	2484(14.9%)	4851(29.1%)	7095(42.6%)	1472(8.8%)	758(4.5%)	3.41
	Experienced teachers	2142(12.9%)	3913(23.5%)	7687(46.1%)	2009(12.1%)	909(5.5%)	3.26
	Experienced in entrepreneurship	2661(16.0%)	4681(28.1%)	6946(41.7%)	1608(9.7%)	764(4.6%)	3.41
	Major fit	2077(12.5%)	3794(22.8%)	7327(44.0%)	2332(13.9%)	1140(6.8%)	3.2
	Trend fit	2398(14.4%)	4845(29.1%)	7104(42.6%)	1518(9.1%)	795(4.8%)	3.39
Entrepreneurship practice	Advisor board	2929(17.6%)	5614(33.7%)	6472(38.8%)	1078(6.5%)	567(3.4%)	3.56
	Exclusive funding	2475(14.9%)	4811(28.9%)	7280(43.7%)	1413(8.5%)	681(4.1%)	3.42
	Integrated service	2918(17.5%)	4828(29.0%)	6596(39.6%)	1533(9.2%)	785(4.7%)	3.45
	Graduates innovation park	2438(14.6%)	4416(26.5%)	7260(43.6%)	1701(10.2%)	845(5.1%)	3.35
	Exclusive practice base	2539(15.2%)	4650(27.9%)	7253(43.5%)	1475(8.9%)	743(4.5%)	3.41
Policy support	Tax exemption	3154(18.9%)	5289(31.7%)	6960(41.8%)	837(5.0%)	420(2.5%)	3.6
	Simplified registration	3082(18.5%)	5180(18.5%)	7085(31.1%)	880(42.5%)	433(5.3%)	3.58
	Initial capital funded	3019(18.1%)	5021(30.1%)	7146(42.9%)	974(5.8%)	500(3.0%)	3.55
	Free training	3012(18.1%)	4778(28.7%)	7237(43.4%)	1102(6.6%)	531(3.2%)	3.52
	Accelerating entrepreneurship	3345(20.1%)	6120(36.7%)	6161(37.0%)	682(4.1%)	352(2.1%)	3.69
	Actual assistance	3391(20.4%)	6029(36.2%)	6256(37.6%)	631(3.8%)	353(2.1%)	3.69

### Entrepreneurship Education Quality Evaluation Scale Output

**FIGURE 1:**
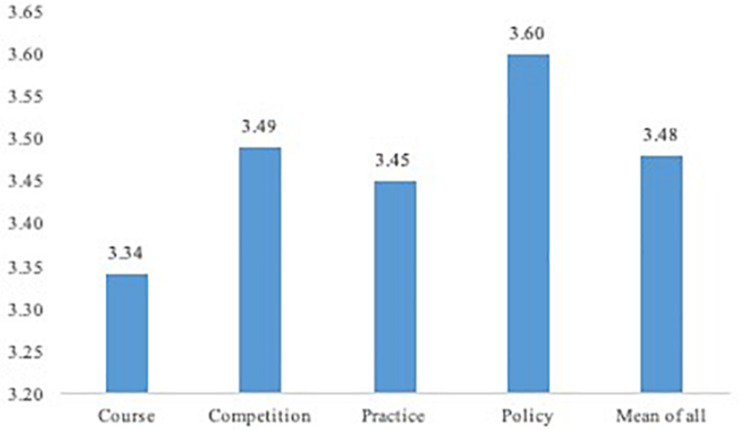
Score of evaluation on entrepreneurship education.

The analysis of each elements and the overall effect of entrepreneurship education were shown in Tables 5–7, as well as Figure 1.

**TABLE 6:** Evaluation of overall effect in entrepreneurship education.

**Primary indicator**	**Secondary indicator**	**Likert point**					**Mean**
		**5**	**4**	**3**	**2**	**1**	
Beneficial effect	Enrich entrepreneurial knowledge	3508 (21.0%)	6192 (37.1%)	6006 (36.0%)	620 (3.7%)	334 (2.0%)	3.72
	Foster a spirit of innovation	3598 (21.6%)	6175 (37.0%)	5960 (35.7%)	592 (3.6%)	335 (2.0%)	3.73
	Improve entrepreneurial skills	3565 (21.4%)	6250 (37.5%)	5947 (35.7%)	578 (3.5%)	320 (1.9%)	3.73
	Inspire entrepreneurial willingness	3565 (21.4%)	6237 (37.4%)	5938 (35.6%)	596 (3.6%)	325 (1.9%)	3.73
	Satisfaction over quality	3133 (18.8%)	5631 (33.8%)	6610 (39.6%)	827 (5.0%)	459 (2.8%)	3.61

**TABLE 7:** Innovation and entrepreneurship course related variables.

**Variables**	**M**	**S**	**1**	**2**	**3**	**4**
Overall satisfaction with course quality	3.61	0.937	1			
Content combined with the trend	3.39	0.997	0.630*	1		
Various types of course	3.26	1.022	0.587*	0.691*	1	
Content combined with expertise	3.20	1.048	0.564*	0.790*	0.674*	1

* Significant correlation at 0.001 level.

According to the results of the frequency analysis and average factor analysis, evaluation regarding the effect of entrepreneurship education was generally positive. The average factor score was 3.48 and the mean of each factor score exceeded 3.34. In comparison, the sampled students rated policy support higher (3.60) and entrepreneurship courses lower (3.34). The average value of “various types of course” is 3.26 and the mode is 3, indicating that the content of entrepreneurship course is scattered and the type is monotone. The overall satisfaction with course quality is moderately related to the other three aspects.

Entrepreneurial course (*x*_1_), subject contest (*x*_2_), entrepreneurship practice (*x*_3_), and policy support (*x*_4_) were taken as independent variables; evaluation of entrepreneurship education was treated as dependent variable (*y*). The equation

$$y=\varepsilon +\beta_{1}\,x_{1}+\beta_{2}\,x_{2}+\beta_{3}\,x_{3}+\beta_{4}\,x_{4}$$

was exploited for stepwise regression analysis in the multiple linear regression analysis. The *adjusted R*^2^ of the model was 0.810 determined by formula

$$R_{a\,d\,j\,u\,s\,t}^{2}=1-\frac{\left(1-R^{2}\right)\,\left(n-1\right)}{n-k-1}$$

where *n* was sample size and *k* was variable quantity. The linear equation responded well to the real data and the response degree was 81.0% (0.810). Durbin-Watson Statistic Value (Durbin and Watson, 1951) was 1.924, which could exclude auto-correlation, and no pseudo-regression was presented. During ANOVA analysis, *F* = 17712.695, *P* < 0.001, indicating that there were significant differences among the four variables. Every VIF was less than 10, which excluded collinearity. Namely, the correlation between the variables was not considered to affect the accuracy of the regression model.

According to (Table 8), the fitting equation was

**TABLE 8:** Multiple linear regression analysis of influential factors.

**Model**	**Statistics model**						
	**Non-standardized coefficient**		**Standardized coefficient**	**t**	**Sig.**	**Collinear statistics**	
					
	**B**	**STD**	**Trail**			**Tolerance**	**VIF**
(Constant)	0.380	0.013	NA	28.658	0	NA	NA
Course	0.081	0.006	0.084	12.990	0	0.275	3.640
Contest	0.330	0.008	0.324	39.606	0	0.170	5.874
Practice	0.123	0.007	0.128	16.407	0	0.189	5.305
Policy	0.801	0.006	0.795	134.597	0	0.327	3.056

*NA, not applied.*

$$y=0.380+0.081\,x_{1}+0.330\,x_{2}+0.123\,x_{3}+0.801\,x_{4}$$

The plus-minus signs indicated the positive/negative influence of each variable and the modulus of its corresponding coefficient represented the extent of its influence. It could be demonstrated that an entrepreneurial curriculum and entrepreneurial practice were negatively correlated to the evaluation, while contest and policy support were positively correlated to the evaluation. Thus, *H1, H2*, *H3*, and *H4* were simultaneously corroborated. The degree of influence from high to low should be ranked as policy support, contest projects, entrepreneurship practice, and entrepreneurship courses.

### Teacher-Student Collaboration Scale Output

The analysis is shown in Table 9.

**TABLE 9:** Frequency analysis of Teacher-student collaboration.

**Primary indicator**	**Secondary indicator**	**Likert point**					**Mean**
		**5**	**4**	**3**	**2**	**1**	
Beneficial effect	Better understanding of the frontiers	3612 (21.7%)	6310 (37.8%)	5324 (31.9%)	902 (5.4%)	512 (3.1%)	3.70
	Enhanced scientific research capability	3639 (21.8%)	6207 (37.2%)	5443 (32.6%)	875 (5.2%)	496 (3.0%)	3.70
	Enhanced capacity for innovation	3670 (22.0%)	6294 (37.8%)	5311 (31.9%)	871 (5.2%)	514 (3.1%)	3.70
	Easy commercialization	3453 (20.7%)	5944 (35.7%)	5786 (34.7%)	953 (5.7%)	524 (3.1%)	3.65

According to the results of frequency analysis and average factor analysis, the evaluation of teacher-student collaboration was generally good with scores of each factor exceeding 3.65, which demonstrated that *H5* was corroborated. However, the evaluation of “easy commercialization” was relatively low, presenting a point of 3.65. It could be interpreted from the analysis that the beneficial effects of teacher-student collaboration were highly recognized, and teachers were expected to play the role of facilitator. It was indicated that students generally believed in necessary hands-on practice, requesting the right to make decisions instead of being a mere vessel to deliver the mentor’s directive. Among the cooperative methods, the most common mode was “student’s innovation under guidance”, which inspired students to spark their subjective initiative rather than being passive performers. Teachers have rich knowledge, which reduce the risk of failure in the process of entrepreneurship. However, there were also obvious problems in the actual operation. “Not being able to get information from the teacher” was one of the major obstacles to jointly carrying out the entrepreneurship project with the teacher.

## Conclusion

From the aforementioned results, the process factors of entrepreneurship education, such as entrepreneurship curriculum, entrepreneurship practice, policy support, and collaboration between teachers and students, collectively demonstrated significance in improving the quality of entrepreneurship education in medical schools. Some process factors presented consistent effects in both medical schools and comprehensive universities, such as teacher-student co-entrepreneurship. This factor has been regarded as a crucial vessel of entrepreneurship education in medical schools (Mcdonnell et al., 2012). According to the survey, collaboration between teachers and students was shown to be particularly useful in understanding the state-of-the-art dynamics of professional knowledge, improving scientific research ability, and enhancing innovation and entrepreneurship ability. Such results were consistent with the situation reported in comprehensive universities (Mcdonnell et al., 2012). The other findings had obviously exhibited different outputs, which have been discussed in the following section.

The most evident finding was that the popularity rate of EE courses in domestic medical schools was rather low and the course design showed a distinctive fragmentation. The results showed that 70.9% of medical schools offered entrepreneurship courses and approximately 60% of sampled students have attended an EE course. These all presented that many of the medical schools in China have realized the significance of innovation and entrepreneurship education. But the results indicated that the entrepreneurship course was mainly conducted as an optional extra. Moreover, it showed that the content of entrepreneurship courses was scattered and the type of course is monotone, which has severely hindered the enthusiasm of medical students in attending innovation and entrepreneurship courses. Although there were also teaching forms such as workshops, interactive seminars, and high-quality lectures, current innovation and entrepreneurship education course designs were still not systematic, which presented a simple patchwork of some fragmented curriculum forms. This was extremely different from the innovation and entrepreneurship education carried out by most of the comprehensive universities. The EE curriculum in comprehensive universities has achieved a full coverage to all the students. The curriculum had a systematic design with rich content and diverse teaching approaches, as Zhang and He (2019) introduced such a curriculum design in Caltech where systematic designs for EE courses have been implemented for years. In addition, the correlation analysis showed that overall satisfaction with entrepreneurship education quality was positively related to entrepreneurship course, among which, “the content is closely combined with the cutting-edge trend,” “The content is closely combined with the professional knowledge,” and “Teaching methods of entrepreneurship courses are various” were highly related. Therefore, improving the course content with the times could help to improve the satisfaction toward education quality.

Entrepreneurship practice has been shown to be able to promote entrepreneurship ability, which was consistent with long-term studies (Drucker, 1985; Spitzeck and Janssen, 2010). Among the relevant elements of entrepreneurship practice, comparing with localized relevant supporting elements, the evaluation of “with exclusive off-campus entrepreneurship practice base” was the lowest. It represented that entrepreneurship practice by medical students was observed to be less open. Namely, they were more inclined to explore practice within campus, which failed to effectively integrate them with the society, market, and economic situation. As an attempt of entrepreneurship practice, entrepreneurship contest features the most typical Chinese educational characteristics. The result of the option survey “Which activity that you have participated in helped you the most” showed that 32.4% of sampled students chose contest as the top prioritized option, which was the most popular option. A previous study (Henkel et al., 2015) has shown that an entrepreneurship contest helpd students to a large extent, which was consistent with the findings of this paper. For the very first time, this study has revealed that the sampled students have a high evaluation on the entrepreneurship contest because of “improvement of team cooperation ability,” “improvement of interpersonal network,” “real entrepreneurial help,” “improvement of entrepreneurial self-confidence,” and “improvement of entrepreneurial ability.” But the evaluation on “easier commercialization of the contest projects” still needed to be improved.

The existing entrepreneurial policies in China do not provide additional support for medical entrepreneurship and the shortage of funds has become the main obstacle for medical students to start their own businesses. National and/or local policies and regulations significantly contributed to the success of medical students’ entrepreneurship, which helped to enhance the entrepreneurial intention. However, for the policy support provided by different stakeholders, the most frequent choice was “tax reduction for graduates established start-ups on national level,” which implied that students held the highest evaluation and expectation for the national policy support. However, judging from the existing policy support for innovation and entrepreneurship, such as *Implementation Opinions of the General Office of the State Council on Deepening the Reform of Innovation and Entrepreneurship Education in Colleges and Universities* and other universal policies targeted at ordinary graduates, there was no additional green channel for medical students to start their own entrepreneurship. Evaluation on “interest-free loans for graduates established start-ups provided by schools” and “free training for graduates established start-ups offered by society” was relatively unsatisfying, which showed that medical students have frequently encountered the difficulty of insufficient funds in the process of entrepreneurship. It was also a problem frequently reported in policy research academic papers. Edoho (2016) reported such a problem in Nigeria, which has seen a throat-cutting competition over national funding under the policy support. It could be seen that the challenge of insufficient funds was much more severe among overpopulated developing countries. Therefore, for medical students, a highly specialized group of students, offering financial support and policies should be an important issue that is paid top priority.

### Implications for Researchers

The theoretical contributions of this paper were mainly presented in three aspects.

First, it filled the blank of the EE evaluation research in medical schools through the empirical study on 76 domestic medical schools. In China, there has been much research on entrepreneurship education, but there were only several studies on the EE evaluation and even fewer on the EE evaluation in medical schools. The research on how to deliver entrepreneurship education, how to evaluate the quality of entrepreneurship education, and how to improve the quality of entrepreneurship education based on the evaluation were relatively weak in China’s medical schools. Scholars have hardly focused on it, which meant the academia naturally lacked convincing research results with strong evidence.

Second, through the data analysis derived from a large sample size and the usage of multiple linear regression analysis methods, this paper revealed that the entrepreneurship education in medical schools was quite different from that in comprehensive universities. The current status and failings for the EE curriculum included its unpopularity, the obvious fragmentation of curriculum design, subpar openness of entrepreneurship practice, and inadequate policy support. The study has provided a practical basis to improve the quality of entrepreneurship education in medical colleges.

Third, this paper used empirical research to test the hypotheses proposed on the basis of a literature review on EE process factors. It clarified the positive significance of the process factors of entrepreneurship education to the quality of entrepreneurship education.

### Implications for Practitioners

The research findings have implications for medical schools that could help them to improve the quality of entrepreneurship education regarding the following three aspects.

First, a personalized and diversified medical entrepreneurship education system should be created to expand benefits for potential audiences. The innovation and entrepreneurship courses should be established with full coverage to all the students in campus, which could involve general EE content such as economics, management, business, and relevant policies and regulations, etc. The lectures are supposed to closely follow the forefront of the era, emphasizing the cultivation of universal awareness and basic knowledge to enhance the popularity of EE courses. Furthermore, the deep integration of entrepreneurship education and professional education should be achieved by the curriculum design underlining subject-oriented professional education with the support of entrepreneurship education elements. Content could be drawn from the Medical school in the University of Michigan, which has systematically designed the course arrangement with the theme of innovative project commercialization at its core. Meanwhile, case studies of medical innovation and entrepreneurship should be conducted to introduce how the contents of EE courses can be integrated with professional knowledge and how EE courses can be implemented based on expertise. Meanwhile, due to the specialty of medical study, students may encounter some market supervision problems such as patent application and transformation and premarket administrative affairs. In the corresponding course design, the administrator of certain government agencies shall be hired to answer questions, expand the knowledge structure of students, and improve the market acumen of students.

Second, the openness of entrepreneurship practice should be strengthened by joint efforts to effectively integrate with the market. In terms of the transformation of entrepreneurship concepts, it is necessary to reverse the on-campus misunderstanding of entrepreneurship cognition and synchronously create an off-campus public opinion delivery. In terms of school internally, it is necessary to break through the shackles of the disciplinary attribute of medical specialty on the concept of entrepreneurship education. Lecturers should also get rid of the misunderstanding of entrepreneurship education, and not that entrepreneurship education is just a narrow sense of “running enterprises.” The core of innovation and entrepreneurship education is to cultivate entrepreneurship spirit. In terms of school externally, medical schools, with their own influence and social appeal, should publicize the importance and social value of medical innovation and entrepreneurship to the entire society, changing the stereotype against medical students. In terms of building a platform, the school should strengthen their cooperation with enterprises. Zhou (2014) emphasized that the combination of Industry-University-Research-Application was actually the combination of education systems and social practice systems. It was an effective way for universities to cultivate innovative and entrepreneurial talents. Enterprises can provide support and services in various aspects such as work placements, target directions, achievement appraisal, and financial assistance. Colleges and universities can transfer high-tech and high-quality talents to enterprises to achieve mutually beneficial situations. It can also be achieved by organizing entrepreneurial teams, integrating laboratories, R&D sections, and incubation parks in the way of school-enterprise cooperation, employing entrepreneurs as practice instructors to give lectures and solve doubts about the difficulties in the practice process, encouraging professionals to join the company to practice, truly contacting the market at zero distance to understand the whole industry chain, so as to improve the ability of teachers to grasp the current trend of the industry, as well as the pertinence and effectiveness of student guidance. In terms of campus culture, by holding versatile extracurricular activities, the campus cultural atmosphere of innovation and entrepreneurship is awakened. For instance, Qian et al. (2019) raised several activities, such as the entrepreneurship salon that encourages students and teachers to brainstorm together, exchange experiences on innovation and entrepreneurship, and excites the spark of entrepreneurship. The workshop enables the design and practice of health products in combination with students’ own specialties.

Third, multi-channel fund support platforms should be constructed to solve the shortage of venture capital. Medical schools should cooperate with the government to build a “policy network” for medical innovation and entrepreneurship. The government should hold joint meetings with medical colleges and relevant functional departments to study and analyze the innovation and entrepreneurship work in medical schools, eliminating departmental barriers, strengthening the link between horizontal departments, building a network of entrepreneurship policies for medical students, and providing assistance and support to start-ups in terms of taxation, financing, and other issues. Advantages of university incubators and influence of the university should be at full function to expand the publicity of innovation and entrepreneurship education, broadening the financing channels and comprehensively attracting social donations and investment. The university-industry interaction should be strengthened, encouraging the entry of industry. Through close cooperation with the industry, it is possible to complement mutual advantages and simultaneously transform the entry of financial capital and venture capital into a conventional mechanism. Finally, alumni should be actively mobilized to back feed alma mater, creating funds and striving for more alumni support and donations.

### Limitations and Research Opportunities

Limitations in the current study have been summarized to offer potential research opportunities for future exploration.

First, this study evaluated the quality of entrepreneurship education in medical schools concentrating on the perspective of students. It might cause inaccuracies as the angle of lecturers was not adequately considered. In future research, all stakeholders related to entrepreneurship education could be taken into account to generate a more comprehensive evaluation.

Second, this study explored several process factors in entrepreneurship education evaluation. It has not covered all the process factors, such as the lecturer teamwork and teaching method. The research scope could possibly be expanded for further research, where all the process factors could be included to conduct an overall evaluation.

## Data Availability Statement

All datasets generated for this study are included in the article/supplementary material.

## Ethics Statement

Ethical review and approval was not required for the study on human participants in accordance with the local legislation and institutional requirements. Written informed consent for participation was not required for this study in accordance with the national legislation and the institutional requirements.

## Author Contributions

All authors listed have made a substantial, direct and intellectual contribution to the work, and approved it for publication.

## Conflict of Interest

The authors declare that the research was conducted in the absence of any commercial or financial relationships that could be construed as a potential conflict of interest.
